# Ultrasound-Guided Bilateral Modified-Thoracoabdominal Nerve Block Through a Perichondral Approach in a Patient Undergoing Bilateral Laparoscopic Inguinal Hernia Repair: A Case Report

**DOI:** 10.2147/LRA.S482038

**Published:** 2024-11-13

**Authors:** Jassim Rauf, Mohammad Mohsin A M Haji

**Affiliations:** 1 Department of Anesthesiology, ICU & Perioperative Medicine, Hamad General Hospital, Hamad Medical Corporation, Doha, Qatar

**Keywords:** modified thoraco-abdominal nerve block via perichondrial approach, laparoscopy, bilateral inguinal hernia, regional anesthesia, levobupivacaine

## Abstract

**Background:**

Modified thoracoabdominal nerve block through the perichondrial approach (M TAPA), described by Tulgar et al in 2019, is a relatively new block. The block is relatively superficial and easy to perform. It has been successfully used in various laparoscopic surgeries and has been shown to reduce the perioperative opioid requirements.

**Case Presentation:**

We report the case of a 41-year-old male with ASA grade 2, who was scheduled to undergo laparoscopic unilateral inguinal hernia repair. The patient had General Anaesthesia with bilateral M TAPA using 30 mL of 0.25% levobupivacaine on each side before the incision. Intraoperatively, the surgeon decided that the patient would require bilateral inguinal hernia repair. The patient received 100 µg fentanyl at induction, and intravenous paracetamol and ketorolac intraoperatively. The surgery was uneventful, and the patient was transferred to a post anaesthesia care unit. In the postoperative period, the patient did not require opioids and received only two doses each of paracetamol and ketorolac. The patient was discharged the following day, without any further sequelae.

**Conclusion:**

When administered bilaterally, MTAPA can provide adequate analgesia and has opioid-sparing effects in both the intra and postoperative periods.

## Introduction

Laparoscopic inguinal hernia repair has grown in popularity in recent years and has become a valid option, especially for bilateral inguinal hernias.^1^ Potential benefits include a decreased incidence of reduced post-operative recovery.^2^ As such, the dermatomal distribution of pain postoperatively might vary; in one study, it was shown that the predominant nerve was the ilioinguinal and iliohypogastric nerve (L1) distribution, which contributed the most to chronic pain.^3^

Regional anaesthesia (RA) has become an essential part of multimodal anaesthesia. It has proven to reduce opioid requirements both intraoperatively and in the postoperative period. Reduced opioid requirements in turn have a positive effect on recovery, early ambulation and discharge from the hospital. However, a recent study demonstrated that the outcomes of RA are influenced by race, insurance and the type of surgery.^4^ An increasing trend to opioid-free anaesthesia, which includes RA, is a promising practice and has proven to be beneficial in terms of patient outcomes.^5^

Modified thoracoabdominal nerve block through a perichondrial approach (M TAPA) was first described by Tulgar et al^6^,^7^ The authors reported a significant opioid sparing effect in their cases.

Our case report is about a patient who received M TAPA for bilateral laparoscopic inguinal hernia repair and its effects on intraoperative and postoperative analgesic requirements.

## Case Presentation

**Figure 1:**
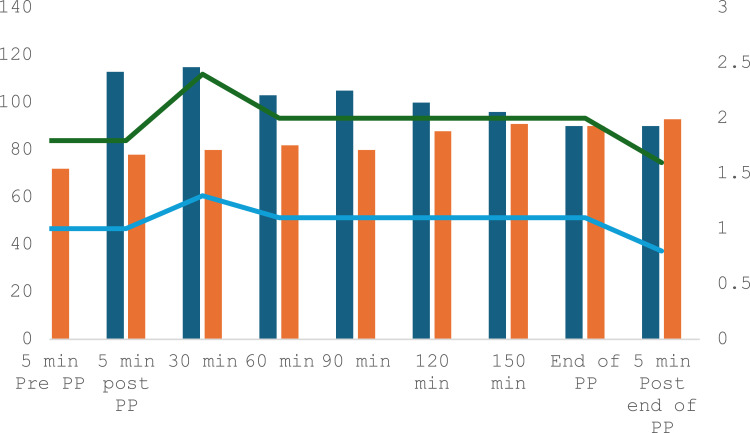
Patient intraoperative parameters from start and end of pneumoperitoneum. Dark Blue Bar: MAP: Mean Arterial Pressure mmHg. Orange Bar: HR: Heart Rate Beats per minute. Green Line: eT Sevo: End Tidal Sevoflurane Concentration %. Blue Line: MAC Sevo: Minimum Alveolar Concentration Sevoflurane %. PP: Pneumoperitoneum. After 5 min post PP all readings are 30 min post PP. Last reading is 5 min post end of PP.

We describe the case of a 41-year-old male (Height 185 cm, Weight 85 Kg) with ASA (American Society of Anaesthesiologists) II, who was scheduled to undergo left-sided laparoscopic inguinal hernia repair. The patient had a history of allergic rhinitis and degenerative disc disease. General anaesthesia with M-TAPA block was planned for this surgery, and the patient consented to the same. On arrival in the operating room (OR), patients were connected to standard AAGBI (The Association of Anesthetists of Great Britain and Ireland) monitoring. After pre-oxygenation for three minutes induction was performed using 70 mg lidocaine, 100mcg fentanyl, 150 mg propofol, and 80 mg rocuronium. Bag mask ventilation was established, and after bag mask ventilation for 2 minutes, the patient was intubated with a regular ETT (Endotracheal Tube) size 7.5 and fixed at 21 cm. Subsequently, the thoracoabdominal area was exposed. The block area was prepared using chlorhexidine. The patient was then scanned with a 6–13-mHz linear probe (Sonosite M-Turbo ultrasound device, Fujifilm, Sonosite, WA, USA) to identifying the 10th costochondral junction (CCJ). The probe was placed in the sagittal direction at the 10^TH^ CCJ. Three muscle layers, the external oblique, internal oblique, and transversus abdominis muscles, and the 10th CCJ were identified. The probe was then slightly angled to visualise the posterior border of the 10^t^ CCJ. A 22 G 80 mm SonoTAP needle was then introduced in a caudad to cephalad direction, aimed at the posterior surface of the CCJ. Once the tip of the needle was confirmed beneath the CCJ, two mls of normal saline was injected to confirm its position. After confirmation, 30 mL of 0.25% levobupivacaine was injected with aspiration every 5 mL. A bilateral block was performed. Intraoperatively, anaesthesia was maintained with 1% sevoflurane minimum alveolar concentration (MAC). After pneumoperitoneum was established, the patient was placed in the reverse Trendelenburg position. Upon exploration, the surgeon decided that the patient would require repair of the bilateral inguinal hernia. The patient did not show any remarkable changes in heart rate (HR) and blood pressure (BP) at the time of incision, except when he was placed in the Trendelenburg position, leading to an increase in BP and a slight increase in HR, which was managed by deepening anaesthesia with increased sevoflurane. In addition, the patient maintained his HR and BP throughout the intraoperative period (Table 1 and Figure 1). Intraoperatively, the patient received 8 mg dexamethasone at induction, paracetamol 1gm, ketorolac 30 mg and ondansetron 30 min before the end of surgery. The patient required additional rocuronium bolus administration. At the end of the surgery, the neuromuscular blockade was reversed with 200 mg sugammadex and after establishing a train of four ratio of more than 1.2 patient was extubated while meeting the full criteria for extubation. The surgery was uneventful, and the patient was transferred to the postanaesthesia care unit (PACU). The patient was monitored in the PACU for 40 min, during which time he was comfortable with an Numerical Rating Scale (NRS) pain score of 0 and thereafter discharged to the ward. Three hours after the surgery, the patient complained of generalised abdominal cramps. However, the patient denied pain at the surgical site or inguinal area. During his stay in the ward, the patient received two doses of 1000 mg of paracetamol and two doses of 30 mg of ketorolac. No morphine was administered intraoperatively in the PACU or ward. The patient was discharged the following day, without any further sequelae.

**Table 1:** Patient Intraoperative Parameters

Time Points	Induction	Post-Block	Incision	Trendelenburg	End of Surgery
BP	137/93 (107)	109/77 (88)	102/79 (87)	142/109 (120)	120/86 (97)
eT Sevo %	0	1.6	1.8	2.4	1.6
HR	76	72	70	80	90

**Abbreviations**: BP, Blood pressure Systolic/Diastolic (Mean Arterial Pressure); eT Sevo %, End Tidal sevoflurane percentage; HR, Heart Rate Beats per minute.

## Discussion

The hallmark of our case report is patients not requiring any intraoperative or postoperative morphine, showing that M TAPA has a significant opioid sparing effect. The dose of fentanyl given, alone, could not have been enough for the entire surgical period and the postoperative period as the half-life of fentanyl is only 45 min. This is the first case report in this surgical population that reported no opioid requirements in the postoperative period and the only analgesics used intraoperatively and postoperatively were paracetamol and ketorolac.

M-TAPA block is an easy to perform block. It is very superficial, the landmark the 10th CCJ is easy to identify, and the needle visualisation is very easy. Till now no complications have been reported. The target area of infiltration is the posterior surface of the 10th CCJ, which lies superficial to the transverse abdominis muscle and away from the abdominal and thoracic cavities. The dermatomal coverage of M TAPA is quite extensive and blocks both anterior and lateral cutaneous branches of the abdominal wall. In one case report, Aikawa et al reported a dermatomal coverage of T3 to T12 and the block duration of 56 hours.^8^ A cadaveric study done by Ciftci et al^9^ demonstrated a dermatomal coverage of T4 to T12. The study demonstrated that the local anaesthetic spread was found over the external oblique and internal oblique muscles and wide area of the transversus abdominis plane.

To date, only one randomised control trial has been published by Alver et al^10^ to evaluate the effects of M-TAPA compared to conventional multimodal analgesia for laparoscopic inguinal hernia repair. In the results of the study, the authors found that the NRS pain score and consumption of tramadol was reduced significantly in those who received M-TAPA block compared to those who were given multimodal analgesia. In conclusion, the authors stated that M-TAPA increased pain recovery scores and provided pain relief to patients. A limitation of their study was that they used the same volume of local anaesthetic in all patients receiving MTAPA. A study by Ciftci et al^9^ on five volunteers, which included two patients who underwent laparoscopic inguinal hernia repair, showed that with different volumes, the dermatomal spread was different. The demographics showed that a smaller volume in taller patients did not achieve the same dermatomal level as a larger volume. Another study done on 10 patients undergoing sleeve gastroplasty showed endorsed these findings. This study conducted by de Oliveira et al^11^ showed that the patients with lesser volume had higher pain scores when compared to higher volume (20 mls on each side 4 patients vs 30 mls on each side 6 patients). The study by Alver et al^10^ may have yielded different results if the volume was adjusted for height. Based on the findings of Citci et al.^9^

M TAPA has also been implicated in other types of surgery. These include gynaecological surgeries and laparoscopic cholecystectomies.^12^,^13^ All studies have shown an over all reduction in postoperative analgesia requirements and opioid sparing effects.

## Conclusion

Our case report shows that M-TAPA can potentially reduce or even diminish opioid requirements intraoperatively, with no opioid requirements in the postoperative period for laparoscopic bilateral inguinal hernia repair. More randomised controlled trials with different local anaesthetic volumes are required to strengthen the evidence regarding the dermatomal coverage and efficacy of this block.
